# Impact and Cost of the HIV/AIDS National Strategic Plan for Mozambique, 2015-2019—Projections with the Spectrum/Goals Model

**DOI:** 10.1371/journal.pone.0142908

**Published:** 2015-11-13

**Authors:** Eline L. Korenromp, Benjamin Gobet, Erika Fazito, Joseph Lara, Lori Bollinger, John Stover

**Affiliations:** 1 Avenir Health, Geneva, Switzerland/Glastonbury, United States of America; 2 UNAIDS, Mozambique country office, Maputo, Mozambique; 3 Mozambique Ministry of Health, Maputo, Mozambique; London School of Hygiene and Tropical Medicine, UNITED KINGDOM

## Abstract

**Introduction:**

Mozambique continues to face a severe HIV epidemic and high cost for its control, largely born by international donors. We assessed feasible targets, likely impact and costs for the 2015–2019 national strategic HIV/AIDS plan (NSP).

**Methods:**

The HIV epidemic and response was modelled in the Spectrum/Goals/Resource Needs dynamical simulation model, separately for North/Center/South regions, fitted to antenatal clinic surveillance data, household and key risk group surveys, program statistics, and financial records. Intervention targets were defined in collaboration with the National AIDS Council, Ministry of Health, technical partners and implementing NGOs, considering existing commitments.

**Results:**

Implementing the NSP to meet existing coverage targets would reduce annual new infections among all ages from 105,000 in 2014 to 78,000 in 2019, and reduce annual HIV/AIDS-related deaths from 80,000 to 56,000. Additional scale-up of prevention interventions targeting high-risk groups, with improved patient retention on ART, could further reduce burden to 65,000 new infections and 51,000 HIV-related deaths in 2019. Program cost would increase from US$ 273 million in 2014, to US$ 433 million in 2019 for ‘Current targets’, or US$ 495 million in 2019 for ‘Accelerated scale-up’. The ‘Accelerated scale-up’ would lower cost per infection averted, due to an enhanced focus on behavioural prevention for high-risk groups. Cost and mortality impact are driven by ART, which accounts for 53% of resource needs in 2019. Infections averted are driven by scale-up of interventions targeting sex work (North, rising epidemic) and voluntary male circumcision (Center & South, generalized epidemics).

**Conclusion:**

The NSP could aim to reduce annual new HIV infections and deaths by 2019 by 30% and 40%, respectively, from 2014 levels. Achieving incidence and mortality reductions corresponding to UNAIDS’ ‘Fast track’ targets will require increased ART coverage and additional behavioural prevention targeting key risk groups.

## Introduction

The HIV epidemic continues to be a significant, if not the foremost, public health and economic and challenge for Mozambique. Significant progress has been made in fighting HIV, but high prevalence persists. Since 2013, UNAIDS and the WHO recommend expanding eligibility for antiretroviral treatment (ART) to a CD4 threshold of 500/uL [1], and in 2015 new evidence emerged that immediate ART enhances both the clinical benefits to patients (morbidity and mortality from HIV/AIDS, and TB) and community prevention effects through reduced HIV infectivity [2,3]. Also voluntary male medical circumcision (VMMC) is recommended since 2007 as an important prevention strategy [4]. In the context of an expanded set of evidence-based control strategies, but a constrained HIV/AIDS budget HIV/AIDS, Mozambique‘s epidemic is entering a phase where strategic balancing and prioritization between interventions, target groups and areas is required to accelerate progress and keep the response financially sustainable.

Mozambique’s national AIDS program started in 2001. Key service delivery areas are condom promotion, community mobilization, HIV counseling and testing (all since program start), prevention targeted at female sex workers (FSW) and clients (since 2006), ART (since 2004), PMTCT (since 2005), VMMC (since 2013). Key implementers include the Ministry of Health, implementing partners of the USA’s PEPFAR program, national NGOs supported by the Global Fund to fight AIDS, Tuberculosis and Malaria (Global Fund), and United Nations agencies and programmes. From 2010 to 2014, the response has been guided by Mozambique’s third HIV/AIDS National Strategic Plan (NSP) [5] and the HIV acceleration plan [5].

At 2014 years-end, the National AIDS Council (NAC) and Ministry of Health, with technical partners including UN agencies, USAID and Civil Society Organizations (CSOs), developed the fourth NSP as a guide for HIV prevention, treatment and mitigation over 2015–2019. UNAIDS and *Avenir Health* provided technical assistance to cost the NSP and estimate its potential impact on the epidemic. Targets for intervention coverage and corresponding expected health impact were set in dialogue among partners, considering existing national health sector targets, desired and feasible targets for additional non-medical interventions,as well as expected domestic and donor resources, based on projections using the Goals [6,7,8,9,10] and Resource Needs Models [11]in the *Spectrum* suite of planning tools.

This paper presents the results of the epidemic and response modelling, in terms of coverage and impact targets for the NSP, as well as resource needs. We review contributions of the respective interventions in three scale-up scenarios, of which the most ambitious was proposed as Mozambique’s official 2015–2019 NSP targets in March 2015, and which is pending approval by the Board of the NAC. Results are discussed in the context of the evolving evidence on ART as a key prevention contributor, the UNAIDS 90-90-90 framework and Fast Track targets for ending the global epidemic by 2030, and a growing recognition of efficiencies, prioritization and increasing domestic contributions needed to sustainably finance national AIDS responses.

## Methods

### Epidemic model

The modelling used the *Goals* model, a dynamic compartmental model, built in the *Spectrum* suite of models, used and validated for projections of epidemic trends and the impact of interventions in several countries as well as for global assessments [6,7,8,9,10].

*Goals* simulates transmission of HIV and its morbidity and mortality consequences for adult populations 15–49 years, who upon reaching a median age at first sex, are allocated into one of five risk categories: stable couples (men and women reporting a single partner in the last year), multiple partners (men and women with more than one partner in the last year), FSW and clients, men who have sex with men (MSM), and injecting drug users (IDU). HIV-infected individuals move through CD4 compartments, which correspond to ART eligibility criteria and mortality patterns. Clinical progression after HIV infection is a function of CD4 count, with associated HIV-related mortality, probability of initiating ART (considering national ART eligibility criteria and coverage levels) and infectiousness.

The impact of behavioural prevention interventions is modelled according to an impact matrix that articulates the impact of each intervention on condom use, numbers of partners and age at first sex in the different risk groups, based on meta-analysis of research studies [12,13,14,15]. The model calculates new HIV infections by sex and risk group as a function of behaviors and epidemiological factors, such as prevalence among partners and stage of infection. The risk of transmission is determined by behaviors (number of partners, contacts per partners, condom use) and biomedical factors (ART use, VMMC, prevalence of other sexually transmitted infections (STIs). Interventions can change any of these factors, and thereby affect the future course of the epidemic.

*Goals* is linked to the *Aids Impact Model* (AIM) module in *Spectrum*, which calculates corresponding epidemic patterns for children (0–14 years) and adults above 49 years. *AIM* also estimates the effects of programs preventing mother-to-child transmission [6,7].

### Biomedical and natural history parameters

*Goals* uses internationally agreed best estimates of biomedical and natural history parameters, such as the risk of HIV transmission per act, and variations in this risk by stage of HIV infection, type of sex act, condom use, etc. By preference, these parameters are kept constant across countries, although certain parameters are allowed to vary somewhat regionally, in order to produce adequate fit. For Mozambique, most parameters were kept constant across the three sub-national models, except for the default HIV transmission probability, and the transmission multiplier effects of STIs and of the primary stage of HIV infection (S1 File).

We assumed that ART reduces infectivity of HIV patients by 80%, as an average effectiveness between relevant recent studies including a 96% reduced infectivity found in a clinical trial across multiple–mainly developed, western–countries with very high adherence [16,17], a 38% reduction in a high-coverage ART program in rural South Africa [18], and 85% virological suppression observed in Swaziland [19,20].

### Model fit of the Mozambique epidemic

For the NSP, *Goals* was fitted to official UNAIDS estimates of Mozambique’s AIDS epidemic up to 2013, which had been produced in *AIM* by statistical fitting of HIV sero-prevalence data from antenatal clinic (ANC) surveillance and national household surveys. Similar to the June 2014 UNAIDS *AIM* estimates, *Goals* modelling was done separately for North, Central and South regions, which were aggregated to produce national result [21].

Demographic, behavioural and epidemiological parameters were quantified based on epidemiological and behavioural data from ANC surveillance [22], program statistics, 2011 DHS [23], 2009 national AIDS Indicator Survey [24], Integrated Bio-Behavioural Surveys (IBBS) of high-risk groups [25,26,27], HIV program statistics (PMTCT by regimen, adult ART, child ART and Cotrimoxazole Prophylactic Therapy), selected population-based research studies [28,29], a Mode-of-Transmission modelling conducted for the 3 regions by UNAIDS [30], and the country’s Global AIDS Response Reports of 2012 and 2014 [31,32] (detailed in S2 File). For community mobilization, the (moderate) effect on reducing multiple partnerships assumed in *Goals* was supported by an effectiveness evaluation of a mass media and community mobilization campaign conducted in 2010 in Mozambique’s four provinces with highest HIV prevalence [33].

### NSP scenarios

The NSP was modelled in three scenarios, differing with respect to coverage levels of key prevention and treatment interventions:

- **Constant coverage / Base-case**: Coverage of all interventions remains constant, at 2014 levels;
- **Current targets:** Scale-up of interventions according to existing targets in the 2013–2015 *HIV Acceleration Plan* [34], 2010–2014 national HIV/AIDS strategic plan [5], the eMTCT plan [35] for 2014−2015, a ‘Concept Note’ grant application submitted by Mozambique in 2014 to the Global Fund for 2015−2017 [36] and National Health Sector Plan 2014–2019 [37] for 2018–19;
- **Accelerated scale-up:** Further increased scale-up, notably for targeted behavioural prevention reaching high-risk groups and other non-biomedical prevention programs, expansion of ART eligibility to include all FSW, and improved patient retention on ART.

Some other interventions were not targeted for scale-up, and kept at a constant coverage across scenarios over 2014–2019 (Table 1).

**Table 1:** Intervention coverage, and corresponding condom usage and partner numbers, in 2019, by NSP scenario and region of Mozambique.

Intervention	*North*			*Central*			*South*			*Sources and definitions for baseline (2014) coverage specified under ‘A*. *Constant coverage’*
	*A*. *Constant cove-rage*	*B*. *Current Targets*	*C*. *Acc*. *scale-up*	*A*. *Constant cove-rage*	*B*. *Current Targets*	*C*. *Acc*. *scale-up*	*A*. *Constant cove-rage*	*B*. *Current Targets*	*C*. *Acc*. *scale-up*	
**General populations: **										
Community mobilization (population covered per year)	19%	30%	30%	7%	15%	15%	17%	30%	30%	[23]; PEPFAR & Global Fund
Mass media (adults reached per year)	49%	49%	80%	57%	57%	80%	48%	48%	80%	
HIV testing and counselling (adults tested per year)	20%	20%	35%	20%	20%	35%	20%	20%	35%	Rapid Diagnostic Test kits used, substracting those for PMTCT & blood bank donations
Condoms promotion & provision (adults reached, incl. social marketing)	8%	27%	27%	20%	36%	36%	27%	36%	36%	Men with > = 2 partners in the last 12 months who reported condom use at last sexual contact [23]
Youth in school reached	3%	3%	20%	3%	3%	20%	3%	3%	20%	
Youth out of school reached	10%	10%	30%	10%	10%	30%	10%	10%	30%	
**Most-at-risk populations, (out-)reached with:**											
FSW & clients: peer education, group discussion, condom provision, IEC	13%	32%	60%	28%	32%	60%	56%	56%	80%	PEPFAR 2013 and Global Fund program data (unpublished)
MSM reached with IEC and lubricants	5.5%	5.5%	23%	6.7%	7%	24%	12.3%	12.3%	33%	PEPFAR 2013 program data (unpublished) United Nations Office on Drugs and Crime, program data for 2014 (unpublished)
IDU: peer education, harm reduction and referral for HIV testing	5%	5%	50%	5%	5%	50%	5%	5%	50%	PEPFAR 2013 program data (unpublished)
**Medical interventions:**											
Males circumcised (15–49 years)	93%	94%	94%	33%	69%	80%	58%	87%	87%	
ART, % of 15+ years adults eligible	56%	75%		64%	81%		57%	85%		Eligible: CD4<350/uL, + all TB/HIV-co-infected + all pregnant women [5,34,36] [37]
+ FSW eligible, from 2015			85%			85%			85%	
Retention on ART, at 3 years after enrolment	52%	52%	70%	52%	52%	70%	52%	52%	70%	National program records [38]
PMTCT: lifelong ART started during or before current pregnancy	51%	93%	95%	56%	93%	95%	66%	93%	95%	
Pediatric ART, % of eligible children	68%	78%	80%	35%	83%	83%	59%	83%	83%	Eligible: all children <60 months & older children with CD4 <350/uL and/or <15%
Condom usage (in % of contacts)*:											
Low-risk contacts	1.7	6.2	11.4	3.4	7.2	11.0	7.3	9.4	14.6	
Medium-risk contacts	6.7	11.9	21.7	12	16	25	25	28	36	
High-risk contacts	44	55	62	50	57	63	50	53	60	
MSM	60	60	62	80	80	81	63	63	65	
Numbers of partners per year*:											
Male: low-risk heterosexual	1			1			1			i.e. those with only one, stable partner
Male: medium-risk heterosexual	3.0	2.9	2.8	1.5	1.5	1.4	3.0	2.9	2.8	i.e. those with multiple partners
Male: high-risk heterosexual	5	4.6	3.7	3.0	2.9	2.4	4.2	4.2	3.5	i.e. FSW clients
MSM	3.0	3.0	3.0	2.5	2.5	2.5	3.0	3.0	3.0	
Female: low-risk heterosexual	1			1			1			i.e. those with only one, stable partner
Female—medium-risk heterosexual	4.0	3.9	3.7	1.5	1.5	1.4	3.0	2.9	2.8	i.e. those with multiple partners
Female—high-risk heterosexual	51	47	38	80	79	64	80	80	66	i.e. FSW

Legend to Table 1: Scenarios: A = constant coverage at 2014 levels; B = current targets; C = Accelerated scale-up (see: Methods).

Interventions kept constant at 2014 proportional coverage across all 3 scenarios are: Peer education in the workplace (3%); cotrimoxazole prophylaxis for HIV-infected children (73%), Transfusion blood units effectively screened for HIV (95%; no health impact modelled); and STI treatment (70%; no health impact modelled).

*Condom usage and numbers of partners are calculated as a function of levels of those behaviours at the 2014 baseline, and the targeted coverage and assumed effectiveness of community mobilization, mass media, HIV testing and counselling, condom promotion, and outreach/behavioural prevention for youth, sex workers, MSM and IDU, according to the Goals impact matrix [13].

Abbreviations: ART = Antiretroviral therapy; FSW = Female Sex Workers; IBBS = Integrated Bio-Behavioural Survey; IEC = Information, Education and Communication; MSM = Men having sex with men; IDU = Intravenous drug users; PMTCT = Prevention of Mother-to-Child Transmission.

### Coverage baselines and targets for the NSP

Coverage of NSP interventions in 2014, was estimated from program records and reports [31,32], surveys in the general population and key risk groups, and selected population-based studies [28,29,30]. Relevant targets up to 2019 were set in dialogue with the NAC, Ministry of Health, technical partners and implementing NGOs, considering existing commitments of official national plans and donor grants (Table 1).

Scenario *‘Current targets’* foresees increasing coverage for community mobilization [33], condom promotion and provision, VMMC, adult and pediatric ART, and PMTCT. ART is scaled-up from 56% to 76% of adults with CD4<350/uL in North region, from 65% to 81% in Center, and from 57% to 85% in South; additionally eligibility includes TB/HIV-co-infected adults and pregnant women (from 2012 and 2014, respectively), in all scenarios irrespective of CD4 count.

Scenario *‘Accelerated scale-up’* additionally scales-up mass media, HIV testing and counselling, behavioural prevention for youth, outreach to MSM (partly through prisons) and IDU, and intensifies outreach to FSW and VMMC. ART is further scaled-up to 85% of eligible PLWH with CD4<350/uL, and all FSW irrespective of CD4 count. In addition, this scenario assumes improving patient retention on ART, from 52% at 3 years after treatment initiation as of 2013 [38] to 70% by 2019, resulting in reduced HIV-related mortality through application of *Goals* survival assumptions [39,40].

Assumptions and targets for coverage of community mobilization reflect weighted averages between the general population and special groups such as miners, long-distance truck drivers and other mobile workers.

Scale-up of condom distribution and promotion considered planned increases in social marketing condoms purchased, and important increases in public distribution. Public-sector distribution (about 21 million condoms in 2014) had remained limited to community interventions distributing male and female condoms to communities and hotspots from district warehouses. Renewed Global Fund support is expected to address this bottleneck, as reflected under ‘Accelerated scale-up’.

Targets for behavioural prevention for high-risk populations reflect the expected feasibility of reaching FSW, MSM and IDU in each region. For outreach to FSW and their clients, the ‘Accelerated scale-up’ target is higher in South (80%) than in North and Center (60%), in view of higher baseline coverage (Table 1). These targets average sub-targets for prisoners in main prisons (80% by 2019), and for miners, truck drivers and other mobile workers, who form part of FSW clients.

For MSM, the overall target reflected sub-targets for MSM as a hidden group, and male prisoners. For ‘hidden’ MSM, the sub-target was limited to 15% for all 3 regions, up from 0.2% in 2014, in line with experiences and views of key implementing NGOs. For prisoners, building on activities implemented by the Ministry of Justice in main prisons, a scale-up from 38% in 2014 to 80% by 2019 is foreseen.

For outreach to IDUs, the ‘Accelerated scale-up’ foresees 50% (3 873 out of 7 746 IDUs) reached with harm reduction and HIV testing and counselling by 2019, against 5.2% (N = 374) in 2014. Considering the criminal status of drug use in Mozambique, needle and syringe exchange and drug substitution were not considered by the NAC and stakeholders.

### Unit Costs

Resource needs for the NSP (in 2014 US $) were estimated by multiplying average service unit costs (Table 2) with annual volumes of service delivery, within the Resource Needs Model (RNM) module of *Spectrum* [11].

**Table 2:** Service delivery unit costs (in US$).

	*2014*	*2015*	*2019*	*Explanation & sources of authors’ estimate*
Community mobilization (per person reached)	6.00	6.00	6.00	Weighted average across unit costs for general population, miners, truck drivers and young girls (unpublished data from Johns Hopkins University and other implementers, and [33]). Reach is defined as completion of 6 sessions; cost includes supervision & monitoring of peer educators and activists. Includes IEC promoting behavioural risk reduction, referral and demand creation for HTC/PMTCT/VMMC, as implemented by grass-root organizations.
Mass media (per person reached)	0.49	0.49	0.49	Inputs provided by the communication working group of the *Conselho Nacional de Combate ao HIV e SIDA* (CNCS). Includes national TV/radio spots and campaigns, novella and movies, and use of social media, at national and community level, in Portuguese and local languages.
HIV testing and counselling (per person tested)	3.34	3.34	3.34	National plans [34,41] and NASA [42]. Includes salaries, reagents and other materials.
Condom promotion & provision (per condom distributed)	0.25	0.25	0.25	[41], [34], Concept Note application submitted to Global Fund, October 2014 [36], programmatic data from PSI, UNFPA, and the *Fundação para Desenvolvimento Comunitária*. It includes procurement of male and female condoms, logistics & distribution (to deposits and communities), and social marketing.
Youth in school (per teacher trained)	87.90	87.90	87.90	Estimates based on inputs from Ministry of Education
Youth out of school—peer education (per youth reached)	4.35	4.35	4.35	Unpublished financial and programmatic data from the HIV prevention program “Geração Biz”
Workplace programs (per person reached)	6.30	6.30	6.30	Concept Note application submitted to Global Fund, October 2014 [36]
Female sex workers (per FSW reached)	39.64	39.64	39.64	
MSM reached with outreach and lubricants (per MSM reached)	64.61	64.61	64.61	
IDU outreach and peer education (IDU reached)	79.86	79.86	79.86	Expenditure analysis by PEPFAR in FY 2013.
STI management (per STI treated)	1.43	1.43	1.43	NASA [42], Concept note application submitted to Global Fund, October 2014 [36]
Transfusion blood safety/screening (per blood pocket screened for HIV)	5.40	5.40	5.40	[34,41], NASA [42], with inputs from Mozambican national Blood Bank
Post-exposure prophylaxis (per PEP kit)	58.28	58.28	58.28	Includes ARVs and logistics [34,43]
Safe medical injection (additional cost for auto-destruct syringes)	0.23	0.23	0.23	Goals/RNM default for Southern/Eastern African region [9,44]
Universal precautions (per hospital bed)	300.00	300.00	300.00	Goals/RNM default for Southern/Eastern African region [9,44]
Male circumcision (per VMMC performed)	104.4	93.43	49.54	Unpublished financial and programmatic data from the VMMC program. The change over time reflects diminishing capital expenditure on infrastructure for VMMC
PMTCT, Counselling—Pre-test (per person reached)	3.97	3.97	3.97	[35,41,43]
PMTCT, Counselling—Post-test (per person reached)	47.35	47.35	47.35	[34,41]. Includes family planning, training of traditional midwives, salaries for nurses and part of the running costs of mobile MCH brigades
PMTCT, Counselling—Post-natal, including breastfeeding) (per mother reached)	53.10	53.10	53.10	[34,41]
PMTCT, HIV test—Mother (per mother tested)	11.00	11.00	11.00	MoH, HIV acceleration plan 2013 [34]
PMTCT, HIV test- PCR for infant after birth (per infant tested)	5.88	5.88	5.88	[41] with a 10% increase to reflect HV-positives retested (6% increase) and waste management (4% increase)
PMTCT, HIV test- Infant after cessation of breastfeeding (per infant tested)	1.32	1.32	1.32	MoH, Health Sector Strategic Plan (2013) [41]
PMTCT, ARVs—nevirapine, for infant (cost per person-day)	0.003	0.003	0.003	[35,41,43]
PMTCT, ARVs—AZT (per person-day)	0.45	0.45	0.45	[35,41,43]
PMTCT, ARVs—Triple treatment (TDF + 3TC + EFV) (per person-day)	0.43	0.43	0.43	[35,41,43]
PMTCT, ARVs—Triple treatment (AZT + 3TC + NVP/EVF) (per person-day)	0.45	0.45	0.45	[35,41,43]
Service delivery (per mother reached)	11.51	11.51	11.51	[35,41,43]
Treatment, First-line ART drugs (adults) (per patient-year)	148.24	148.24	148.24	[35,41,43]
Treatment, Second-line ART drugs (adults) (per patient-year)	389.48	389.48	389.48	[35,41,43]
Treatment, Laboratory tests for patients on ART (per patient-year)	40.58	40.58	76.31	[35,41,43]. Includes CD4 and VL. Also includes TB diagnostic, i.e. Smear test (75% of patients, twice yearly), LED Microscopy (25% of patients, twice year), Chest X-ray (8% of patients yearly), Culture & DST 1st Line (25% of patients yearly) and Xpert (18% of patients yearly)–for an average $ 16.4 per adult-year. Increase in 2019 reflects adoption of viral load testing, at 1 test per patient per year by 2019, through an exponential growth starting from 5% in 2015.
Treatment, Cotrimoxazole prophylaxis (per patient-year)	27.88	27.88	27.88	MoH, Health Sector Strategic Plan (2013) [41]
Treatment, TB prophylaxis (per patient-year)	1.26	1.26	1.26	[41]
Treatment, Nutrition supplements in first six months of ART (per patient-year)	17.30	17.30	17.30	Concept Note application submitted to Global Fund, October 2014 [36]
Treatment, ARV drugs, children (per patient-year)	124.48	124.48	124.48	[35,41,43]
Laboratory tests for child on ART (per patient-year)	29.00	29.00	29.00	[41]
ART delivery including treatment of opportunistic infections (per patient-year)	16.62	16.62	16.62	[41]
**Policy and Program Support (national annual total, in US$ million per year):**				
National Program coordination	4.14M	4.14M	4.14M	Expenditures from 2011 [42]
Monitoring and Evaluation	18.81M	19.08M	17.77M	[41,42] includes epidemiological surveillance, Information Technology & data entry equipment, data collection instrument, registers printing and supervision
Training	5.00M	5.33M	2.49M	[41,42]. Includes training of health care workers, lay counselors and peer educators (with an up-front training investment during roll-out of PMTCT Option B+).
Health systems strengthening	20.00M	27.99M	25.74M	Extrapolation from estimates of [41]. Includes construction /rehabilitation of health units and warehouses, and acquisition of equipment and vehicles.
Community systems strengthening	2.70M	2.70M	4.09M	Concept Note application submitted to Global Fund, October 2014 [36]; and inputs from members of the Mozambican Civil Society Platform.
Orphans & Vulnerable Children	10.84M	11.88M	14.80M	Implementing partners and [41,42]
Home-based care	7.50M	7.85M	7.45M	Implementing partners and [41,42]
Human Rights and Gender	0	0.34M	3.62M	Implementing partners, following UNAIDS guidelines for human rights programs in the areas: stigma reduction, legal services, law review and reform, legal literacy, training of health care providers, sensitization of law enforcement agents and women’s rights in the context of HIV) [41].

Legend to Table 2: All values in stated in $ US, using a MZN conversion factor of 32 over 2014–2019. Unless indicated, unit costs are assumed to be the same across the 3 regions; changes from 2014 to 2019 in unit costs are linear, unless indicated. For costs estimated from PEPFAR expenditures, unit costs excluded USG program management and overheads. Abbreviations: ART = Antiretroviral therapy; ARV = antiretroviral; AZT = azithromycin; FSW = Female Sex Workers; IBBS = Integrated Bio-Behavioural Survey; IEC = Information, Education and Communication; MSM = Men having sex with men; NASA = National AIDS Spending Assessment; IDU = Intravenous drug users; NASA = National AIDS Spending Assessment; PMTCT = Prevention of Mother-to-Child Transmission; NVP = nevirapine; TDF = tenofovir; 3TC = lamivudine; EFV = efavirenz.

Where possible, service unit costs were extracted from cost estimates from national HIV/AIDS programs [34,35,37,41].When information was not available, these were estimated from financial records and programmatic data of key implementers including unpublished expenditure analysis by PEPFAR, the 2010–2011 National AIDS Spending Assessment [45], the concept note application submitted to the Global Fund in October 2014 [36], and data from implementing NGOs. Final estimates were agreed with implementing partners, assembled in a working group convened by UNAIDS.

Costs of ARVs, laboratory tests and other essential commodities refer to actual procurements in Mozambique and were taken from the US Government/ PEPFAR Supply Chain Management System [43]. Price changes over 2014–2019 for first-line and second-line ARVs were estimated by extrapolation of trends in PEPFAR ARV procurements globally, giving a 16% reduction in unit price from 2015 to 2019. Laboratory cost per adult patient-year (including TB diagnosis) was assumed to increase from US$ 40.6 in 2014 to US$ 76.3 by 2019, reflecting increasing use of viral load testing (at US$ 30 per patient per year–i.e. without considering pending price reductions for viral load tests).

Costs of essential commodities are inclusive of service delivery and supply chain costs, at a fixed 17.5% [34]. For health sector-based services (ART, PMTCT and Testing & Counselling), human resource costs were integrated in the respective unit costs through a normative bottom-up approach, assuming a fixed health worker time per service unit and fixed health worker capacity.

For simplicity, unit costs were kept fixed over 2014–2019 for most other interventions and budget items. This likely overestimates costs for interventions with falling commodity prices and/or which are likely to achieve economies of scale or efficiencies with program maturation. An exception was made for VMMC, for which cost per man circumcised was assumed to fall from US$ 104 in 2014 to US$ 50 by 2019, reflecting an expected reduction in investments needed in infrastructure and devices. The $ 104 was obtained from PEPFAR, Mozambique’s main VMMC funder and implementing partner. Of this baseline cost, 43% covered capital investment (health unit rehabilitation and construction) and program management, which progressively reduced to 0 by 2019. Furthermore, the ‘Accelerated scale-up’ scenario assumed another US$ 10 unit cost reduction from transitioning to a cheaper VMMC device.

For ART, service delivery unit costs were kept constant. The additional cost of improving patient retention under ‘Accelerated scale-up’ was not explicitly modelled, but reflected under increasing cost of second-line ARVs (for patients who failed first-line ARVs, accumulating with years on treatment), which are more expensive than first-line ARVs.

Program support costs were determined based on actual expenditures (national program coordination), extrapolated from existing plans and identified needs (M&E, Training, Health & Community Systems Strengthening). Since these costs do not increase proportionally with intervention scale-up, an annual US$ 48 million was assumed throughout 2014–2019, in all scenarios.

Mitigation includes support to orphans and vulnerable children (OVCs), HIV-related human rights and gender programs and home-based care. OVC was costed as a lump sum, allocated across regions by their numbers of PLWH. The nation-wide lump sum was based on the price of support kits provided to OVCs by the Ministry of Gender and Social Affairs, which is consistent with unit expenditures on OVC by US Government implementing partners multiplied with OVC numbers (from 2012 and 2013 program data) and a targeted 70% coverage nation-wide by 2019.

For HIV-related human right and gender programs, no package of services was yet defined for Mozambique. These were costed using a normative approach for intervention activities and budgets. Activities were aligned to seven programs (stigma reduction, legal services, law review and reform, legal literacy, training of health care providers, sensitization of law enforcement agents and women’s rights in the context of HIV) recommended by the UNAIDS Human Rights Reference Group [46].

For home-based care, unit cost corresponded to PEPFAR recurrent expenditures (excluding training and program management), multiplied with 34% coverage of known PLWH according to 2013 programmatic data, and assuming a gradual reduction to 22% by 2017, to reflect decreasing need for home-based patient care provided by community organizations following expansion of facility-based ART. The resulting national lump sum was allocated across regions proportional to their ART patient numbers in 2014.

### Outcomes considered

NSP scenarios were evaluated based on effects on HIV incidence and new infections, and HIV/AIDS-related mortality rate and numbers of deaths, focusing on adults 15–49 years. For costs per infection and per death averted, in contrast, infections and deaths among all ages are considered. For these cost-effectiveness metrics, both costs and health impacts were discounted at 3% per year. Cost savings (through ART prevented and health care needs postponed) were considered within the 2015–2019 horizon.

Prevention contributions (in terms of infections averted) of individual interventions were calculated by keeping coverage constant at 2014 level for one intervention at a time, while maintaining scale-up for all other interventions as in ‘Accelerated scale-up’ scenario.

## Results

### Fitted sub-national epidemics, up to 2014

**Fig 1:**
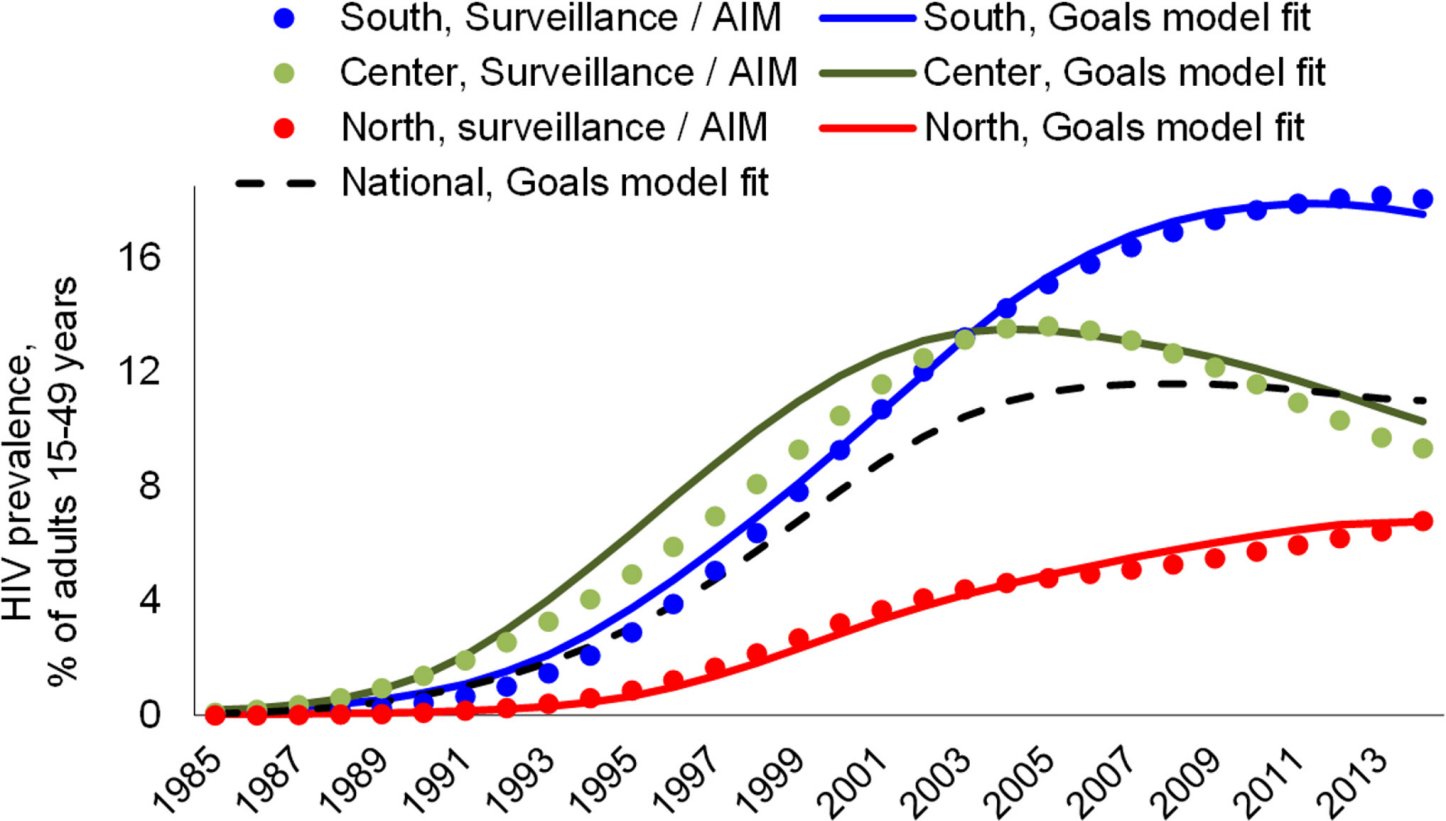
*Goals* model fit to historical HIV prevalence trends, 3 regions of Mozambique. Surveillance/AIM represents the statistical estimates of epidemic trends as of 2014 using the Spectrum/AIM version 5.1, beta 34.

With the behavioural, biomedical and natural history quantifications specified (methods and S1 and S2 Files), *Goals* closely fitted HIV sero-prevalence trends over 1985 to 2013 for all three regions (Fig 1). To achieve this, condom usage was adjusted downward and numbers of partners adjusted upward compared to survey responses, which is justified in view of suspected social desirability bias, due to which respondents often over-report condom usage and under-report partner numbers. HIV prevalence has risen highest in Southern region (which is most developed, and neighbors South Africa, where many Mozambican workers migrate); is moderately high but with a strong reversal and ongoing decline in Center region, and lower but rising in the more remote North.

### Behavioural risk reductions

As a result of scale-up of behavioural prevention interventions, *Goals* estimates marked increases in condom use among all risk groups, in all regions but most notably in the North (Table 1). Based on these projected condom usage rates, the NAC proposed as behavioural outcome target for the NSP to increase condom usage among people with two or more partners (including commercial partners/sex work) by 50% from the 2014 level (of 12%).

Similarly, intervention scale-up is projected to reduce numbers of partners, most notably for medium-risk groups and secondarily for high-risk groups.

### HIV incidence and mortality under NSP scenarios

**Fig 2:**
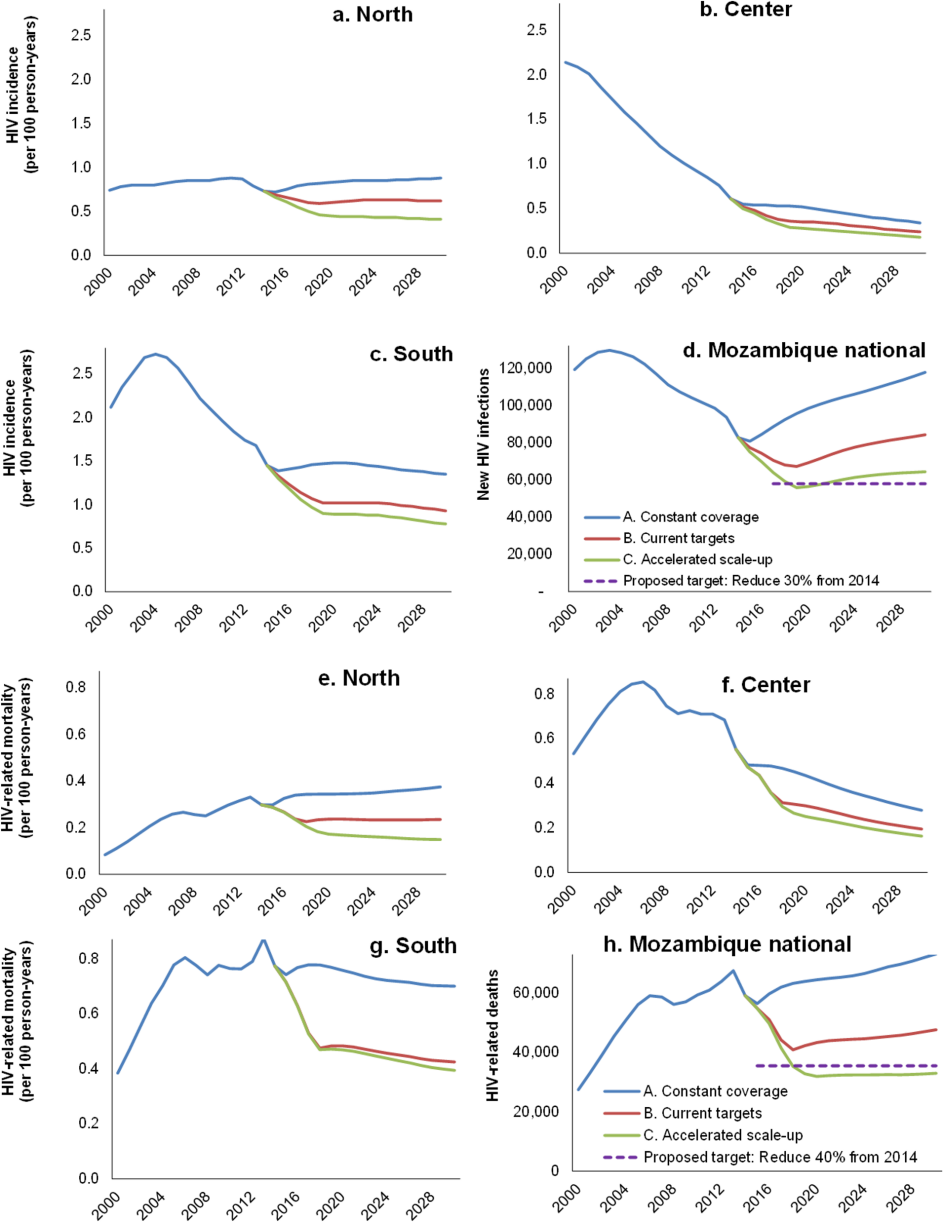
HIV incidence rate by region of Mozambique (a-c) and national total annual new HIV infections (d); HIV-related mortality rate by region of Mozambique (e-g) and national total annual HIV-related deaths, in three NSP scenarios, each for adults 15–49 years.

Under ‘Constant coverage’, the *Goals* model projects a fairly stable HIV incidence over 2015–2019, close to rates in 2014, in all 3 regions (Fig 2A–2C). In the advanced epidemics in Southern and Center regions, this stable incidence follows a progressive decline from peaks around and before 2003, respectively. In the North, incidence is stable at a level similar to that since 2002, although with a slight temporary decline around 2014–2015 which follows rapid ART scale-up over 2013–2014.

Given population growth, these incidence patterns combine into a progressive rise in annual HIV infections nation-wide, from 2015 as a temporary minimum, back to the level of 2012 by 2019 (Fig 2D).

Program scale-up under ‘Current targets’ reduces new infections considerably, reaching a low around 2019, two years after reaching 2017 ART targets. Slight further scale-up of prevention interventions over 2018–2019 does not entirely prevent a subsequent rise in annual infections, which approaches 2014 levels again by 2028. Only ‘Accelerated scale-up’ sustainably reduces annual infections to 30% below the 2014 baseline by 2019, with a fairly stable annual number also over next years.

Current targets will reduce annual new infections (among all ages) from 105,000 in 2014 to 78,000 (19% reduction) in 2019. Accelerated scale-up will reduce annual new infections to 65,000, 33% below the 2014 level.

Similar patterns are apparent for HIV-related deaths. Under ‘Constant coverage’, mortality is fairly stable over 2015–2019 (slightly rising in low-prevalence North; slightly decreasing in the advanced epidemics with higher ART coverage of Center and South; Fig 2E–2G). As a result, annual deaths continue a slow increase as before 2014 (Fig 2H). Temporary mortality fluctuations (e.g. dips in 2007 and 2014, and a peak in 2013) reflect strong, immediate effects of recent ART scale-up, whose impact in individual patient peaks 1–2 years following treatment initiation, followed however by mortality resurgence over subsequent years, if fewer new patients start ART while patients enrolled earlier begin failing treatment or get lost. Nation-wide, ‘Current targets’ will reduce deaths (among all ages) from 80,000 in 2014 to 56,000 (a 33% reduction) by 2019.

‘Accelerated scale-up’ will reduce all-age deaths to 51,000 in 2019, 45% below the 2014 number. Over 2015–2019, this scenario averts 125,000 deaths, of which 12,000 through improved patient retention on ART.

### Contribution of risk groups and interventions

**Fig 3:**
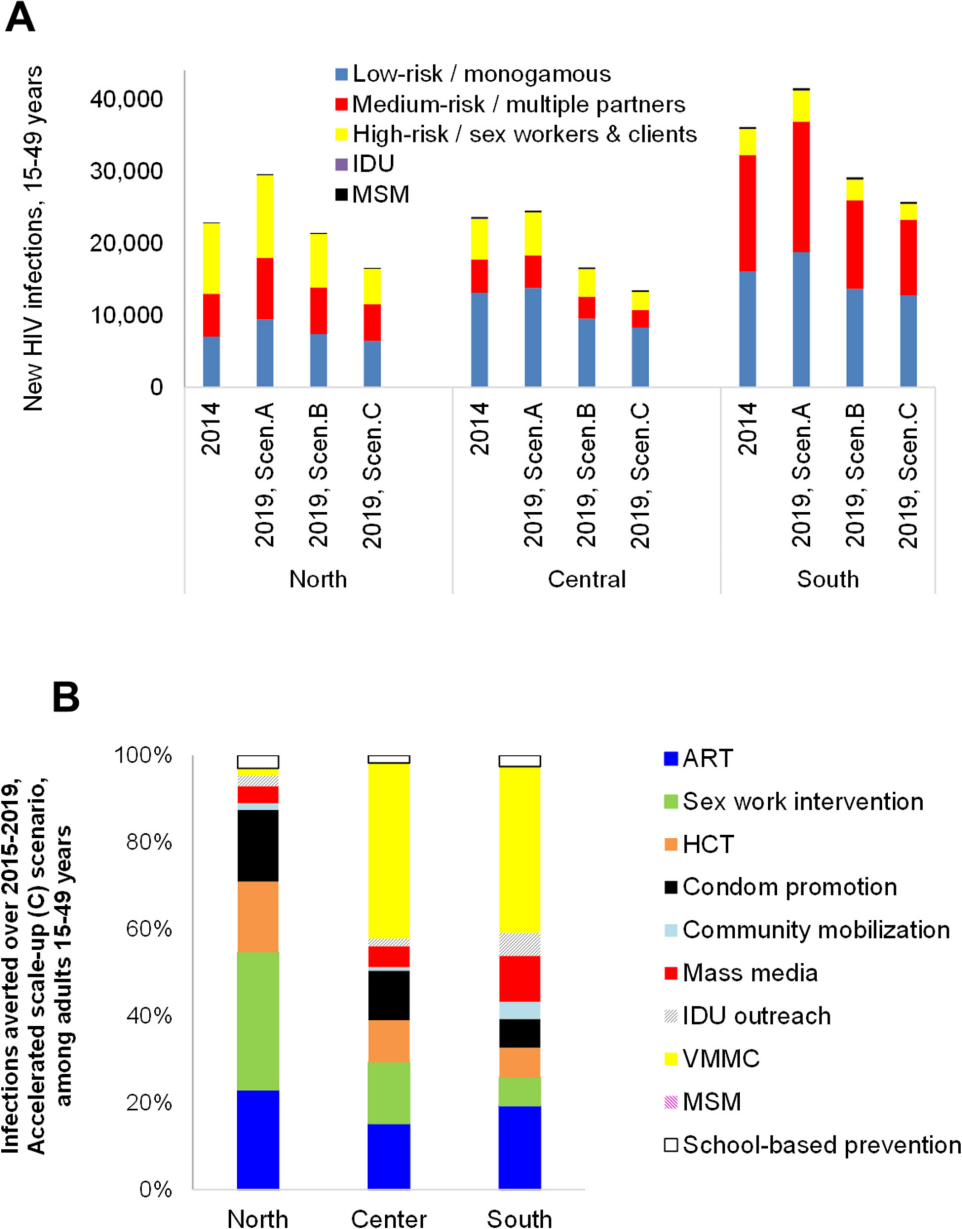
(a) New HIV infections among 15–49 years old adults by risk group, scenario and region, compared to 2014 baseline; (b) HIV infections averted thanks to incremental intervention scale-up over 2015–2019, compared to a scenario with coverage constant at 2014 levels, by region in the ‘Accelerated scale-up’ (C) scenario, adults 15-49-years.

Up to two-thirds of new infections occur in adults who have multiple partners and/or who engage in commercial sex. Especially in Northern region, most new infections continue to occur in these higher-risk groups (Fig 3A), and NSP scenarios avert most new infections in these higher-risk groups.

Comparing infections averted among interventions, in the North, behavioural/prevention outreach to sex workers and their clients, and condom promotion (which reduces risk among people with multiple partners) are key contributors to infections averted over 2015–2019 (Fig 3B). In Center and especially South, with comparatively higher prevalences in the general population, VMMC (from baseline levels lower than in North), and mass media are relatively more important. ART scale-up is a key contributor to infections averted in all three regions.

### Resource needs

**Fig 4:**
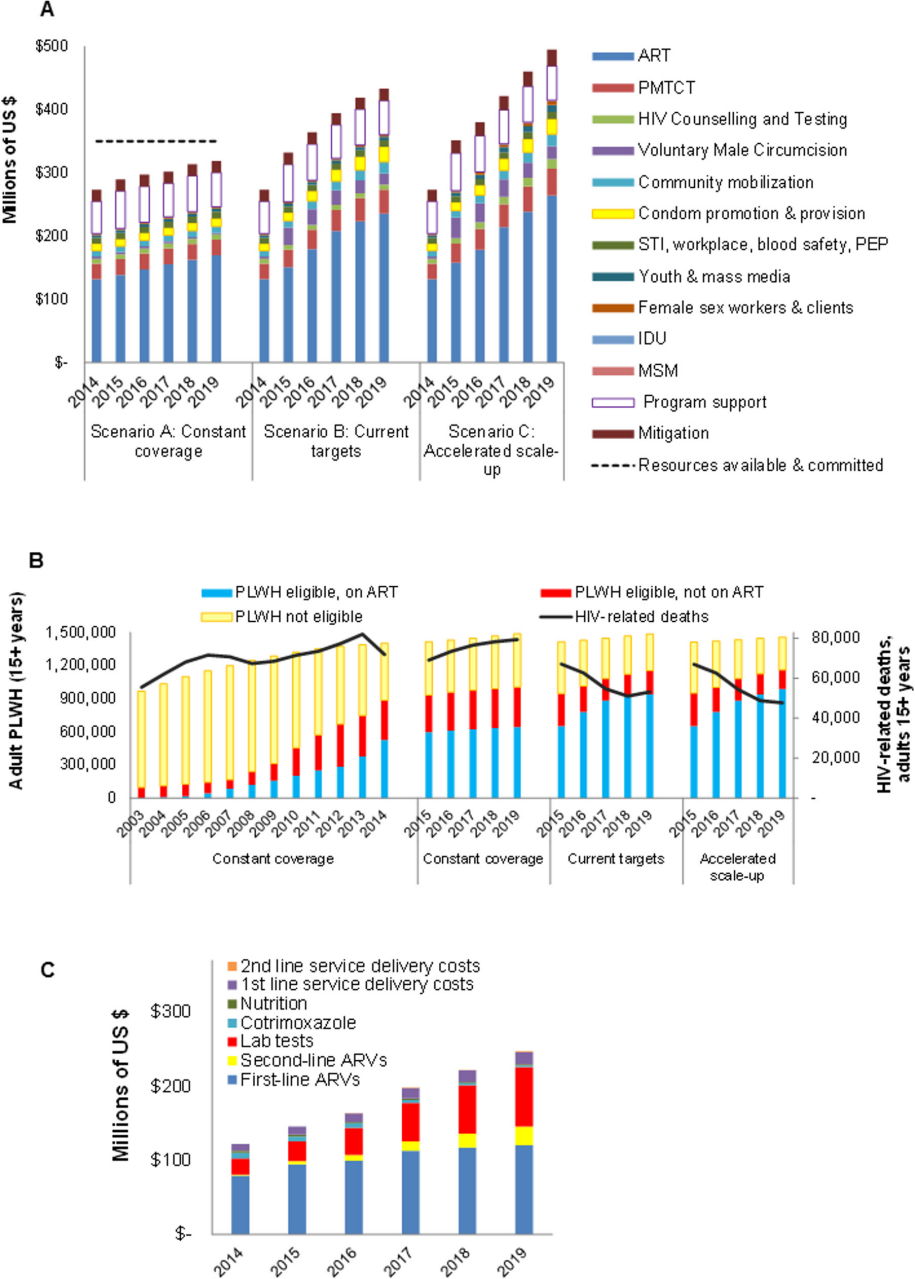
Costs and cost drivers of the Mozambique NSP 2015–2019: a) Resource needs, by scenario and intervention; b) PLWH on first-line and second-line ART, ‘Accelerated scale-up’ scenario; c) ART cost break-down, ‘Accelerated scale-up’ scenario. In (a), the dashed line represents resources available and committed from within the national government, other Mozambican implementers, the Global Fund, PEPFAR and other donors–as of January 2015.

From US$ 273 million program cost in 2014, NSP scenarios require increased resources, to US$ 319 million in 2019 under constant coverage (due to population growth, and increasing need of ART and PMTCT as PLWH progress clinically), US$ 433 million for ‘Current targets’, or US$ 495 million for ‘Accelerated scale-up’ (Fig 4A).

The largest expenditure components would be ART (53% in scenario C in 2019), Program Support (11%), PMTCT (9%, when counting lifelong ART for pregnant women under ART), mitigation (5.2%), Condom promotion and distribution (4.9%), VMMC (4.2%), Community Mobilization (3.7%), and HIV testing and counselling (3.1%).

Cost increases from 2015 to 2019 largely reflect increasing numbers on ART (Fig 4B), including an increasing need for second-line ARVs (Fig 4C) for an estimated 0.65% of ART patients in 2014 up to 5.1% of patients in 2019.

### Cost per infection or death averted

Compared to constant coverage, scale-up to current targets will avert 110,000 infections over 2015–2019, whereas Accelerated scale-up would avert 145,000 infections (discounted, across all ages). This corresponds to a discounted cost per infection averted of US$ 16,612 (Current targets) or US$ 13,380 (Accelerated scale-up).

Discounted costs per death averted (across all ages) are higher (US$ 18,966 and US$ 17,377, respectively), since over 2015–2019 the NSP will avert fewer deaths than infections (96,000 deaths under ‘Current coverage’, and 114,000 deaths under ‘Accelerated scale-up’). Accelerated scale-up lowers costs per infection and death averted compared to ‘Current targets’, thanks to an enhanced focus on behavioural prevention for high-risk groups, which saves many infections and deaths at relatively low cost.

**Fig 5:**
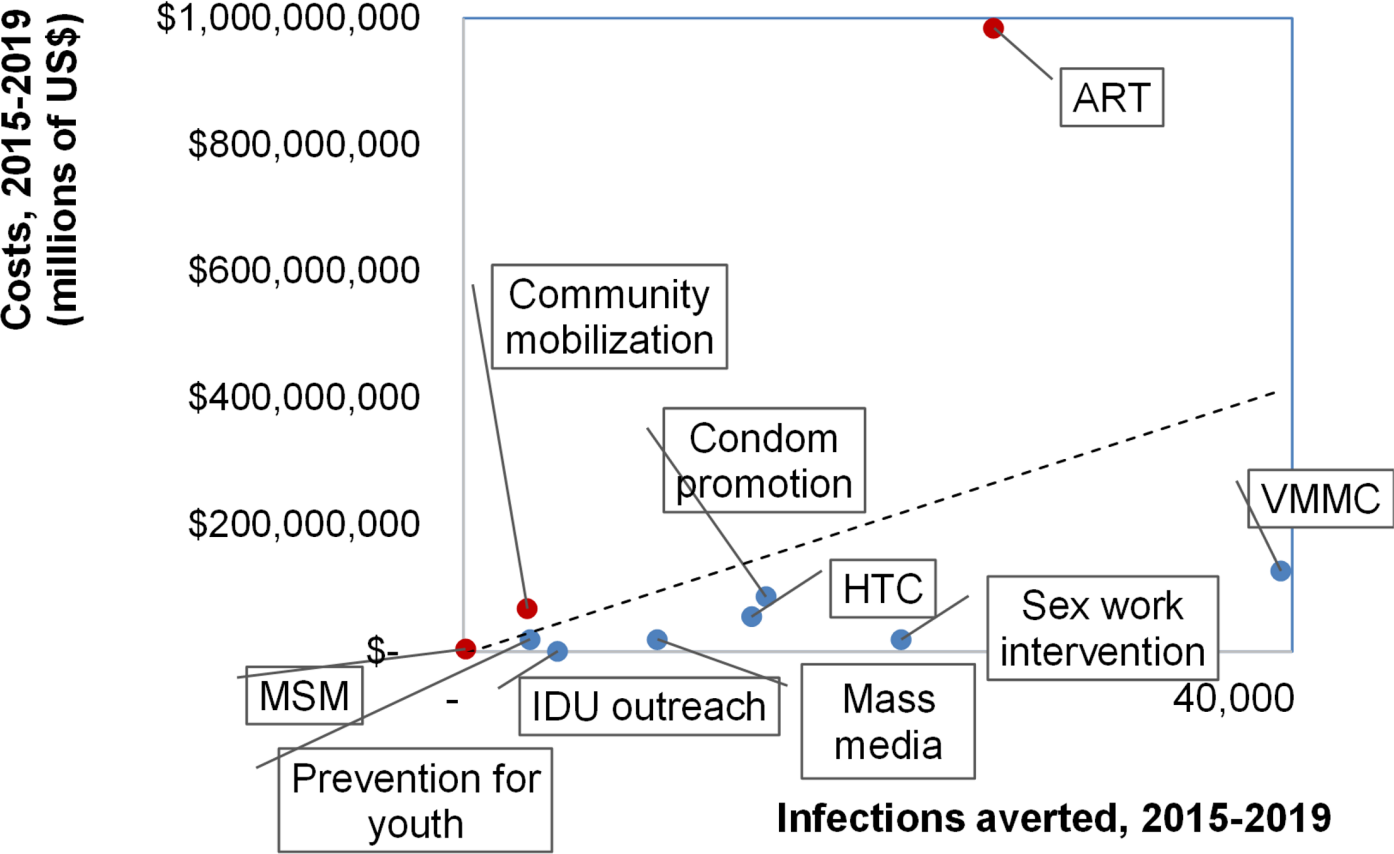
Infections averted (among all ages) from coverage scale-up over 2015–2019, relative to resource needs over 2015–2019, by intervention, in the ‘Accelerated scale-up’ scenario. Both costs and infections averted are discounted, at 3% per year. Abbreviations in Fig 5: ART = Antiretroviral therapy; Sex work = behavioural prevention for Female Sex Workers and their clients; HTC = HIV Testing and Counselling; MSM = Men having sex with men; IDU = Intravenous drug users; PMTCT = Prevention of Mother-to-Child Transmission; VMMC = voluntary medical male circumcision; Youth = behavioural prevention for youth in and out of schools.

Among interventions, scale-up of prevention outreach for sex workers and IDU, mass media, and HIV testing and counselling save relatively many infections at low cost (Fig 5) in all regions. Scaling-up condom promotion and school-based prevention are also relatively cost-effective. Costs per infection averted are higher for community mobilization and outreach to MSM. ART scale-up to above 2014 levels saves many additional infections but at high cost, so is relatively costly per infection averted. Nation-wide, VMMC scale-up averts most additional infections, at low incremental cost in all regions despite varying baseline circumcision prevalences.

## Discussion

Mozambique’s proposed NSP, supported by an evidence-based epidemiological projection, represents a sensible next phase in the country’s AIDS response. If coverage targets as adopted for ‘Accelerated scale-up’ are reached, Mozambique could feasibly expect to reduce new infections by 30–33%, and deaths by 40–45%, in 2019 compared to 2014 levels. The NSP’s model-based target setting builds on previous work but benefits from more and better data, particularly on local intervention unit costs and epidemic dynamics, and national policy analysts used a more integrated set of tools linking interventions with cost and impact, than ever before.

A formal cost-effectiveness analysis–which should consider not only costs but also financial savings from health care averted or postponed beyond 2019 –was beyond the scope of this analysis. Presented incremental costs per infection or death prevented (over 2015–2019 alone, relative to a scenario with coverage maintained at 2014 levels) are in the range of values generally considered cost-effective for countries with incomes similar to Mozambique, with a GDP *per capita* around 1,100 international dollars over 2010–2014 [47,48,49]. In addition, infections averted bring future savings as people will not need treatment. ART costs per patient, combined with expected survival and a 3% annual discount, imply a net present value of the lifetime cost of treatment of about US$ 3,100. Each infection averted within 2015–2019 produces these savings, which partially off-set costs of scaling-up behavioural prevention. Other future costs averted (for example, orphan support) will offset even more of the needed investments. Despite the simplicity of our cost-related outcomes, the comparison of NSP scenarios illustrated the good value for money of program packages prioritizing prevention for key groups–leading the NAC and partners to, for the first time, recognize these as key complements to existing targets for biomedical interventions.

While NSP modelling and target setting was undertaken separately for the three regions with their varying epidemics and baseline service coverage (notably for behavioural prevention for FSW and clients), final coverage targets came out similar across regions. As a result, impact contributions from *incremental* scale-up of the respective interventions, when compared to their 2014 baseline levels, differed somewhat across regions. Up to 2014, male circumcision through traditional practice as was prevalent before scale-up of VMMC was already preventing many infections in Northern region (not reflected in the VMMC shares in Fig 3B), whereas behavioural prevention for sex workers and clients already prevented many infections in Southern region with its advanced epidemic and response.

Nevertheless, country-wide the proposed NSP coverage targets for ‘Accelerated scale-up’ seem a sensible and rational balance between behavioural and biomedical interventions, which should consolidate incidence and mortality reductions started recently, and enhance value for money and sustainability of the national AIDS response. The NSP projections also form a basis for further refined prioritization tailored to regions’ evolving epidemic, response and service costs.

NSP targets for behavioural prevention in key groups (except for marginalized, difficult to reach IDU and MSM) as well as outreach to youth, VMMC, HTC and PMTCT, are in line with global Fast Track targets. The 2015–2019 NSP as proposed is an important step toward reaching the global goal of ending AIDS by 2030. Yet, targets defined for Accelerated scale-up will not permit reaching the Fast Track target of reducing new HIV infections by 80% over 2010−2020 and by 90% over 2010−2030 [50]).To reduce new infections by more than 33% by 2019 and further thereafter, requires a more aggressive program package.

Expanding ART to PLWH with CD4 between 350-500/uL and/or sero-discordant couples, according to curren WHO recommendations [1] or to all PLWH asunder UNAIDS’ 90-90-90 targets (90% of PLWH diagnosed, of whom 90% enrolled and retained on ART, with 90% adherent [50]) would greatly enhance population-level impact. However, unlike higher-income countries in Southern and Eastern Africa, the Mozambican Government has not yet considered such treatment expansion, given resource and system constraints. From current prevalence trends, that implies over 1.5 million PLWH on ART by 2019 i.e. 600,000 more than in the Accelerated scale-up scenario–which at a gross US$ 230 per patient-year would require an additional US$ 138 million annually (28% above ‘Accelerated Scale-up’).

Reaching ‘Fast track’ targets could also be supported by intensifying behavioural prevention efforts among highest-risk groups, further or quicker scale-up of VMMC in Center and South (if judged feasible), and/or higher coverage and/or smarter targeting to youth–especially girls–in and out of schools.

Reaching targets for ‘Accelerated scale-up’ will, in 2019 alone, require US$ 130 million more than the US$ 350 million available for HIV in Mozambique in 2014 [32]. Increasing commitment of PEPFAR for fiscal year 2016, along with the Global Fund concept note grant signed in June 2015, possible additional Global Fund funding from 2017, and an increased state budget allocation for HIV in 2015 [36] could enlarge the resource envelope to US$ 400–450 million per year for 2015–2017. The remaining gap could be closed with additional government expenditure on health (8% only in 2012 [51]) and increased budget allocation from the Health Ministry for HIV, estimated at just 10% in 2013 [36], especially as absolute government revenues are expected to increase. Reaching ‘Fast track’ targets would require additional resources which Mozambique’s economic growth may not be able to cover, and other innovative funding mechanisms need to be considered.

These findings should be interpreted in the context of certain limitations. The model was structured and quantified on available evidence from Mozambique and worldwide. However, sexual mixing between risk groups is limited in the *Goals* model, which may cause over-estimation of intervention impacts on new infections.

The projected mortality impact is driven by ART, modelled based on Southern Africa region-wide survival patterns, with mortality adjusted upward by 20% to account for relatively poor patient retention in Mozambique [52]. This effectiveness remains to be validated against longitudinal patient cohorts and/or population-based data from Mozambique, as in recent mortality impact assessments by neighboring countries [53,54]. Similarly, Mozambique lacks population-based data to validate assumptions on effectiveness of ART in reducing HIV incidence, nevertheless a significant contributor to infections averted over 2015–2019. Assumptions on mortality and transmission impacts of ART in Mozambique are more conservative than in earlier models of global and African country epidemics, reflecting relatively low patient retention on ART in Mozambique [38,52]. Nevertheless, the assumed 80% reduction in infectivity on ART (across scenarios) is higher than the 70% assumed by UNAIDS in its 2015 estimation of national HIV burdens and trends across low- and middle-income countries.

For preventive interventions, *Goals* may under-estimate impact especially in longer-term, as it does not fully capture the dynamics of how reduced risk behaviours impact HIV transmission both directly and indirectly by lowering prevalence of cofactor STIs [55]. *Goals* may also under-appreciate MSM interventions, because MSM are assumed to not have non-marital heterosexual relationships–which may not apply in Mozambique. Lacking an age stratification in coverage, *Goals* projections assume VMMC to be homogeneously distributed through the male population 15–49 years. If VMMC preferentially reaches young men–per Mozambique’s program policy and targets–impact may be different: e.g. greater in the long-term, but possibly less in the short-term, when boys circumcised have not yet started high-risk behaviours.

In costing, not all unit costs or cost components corresponded to observed expenditures (e.g. for human rights programs). For health sector-based services, human resource costs assumed fixed health worker time per service unit and fixed health worker capacity, without considering possible inefficiencies in health worker performance. High unit costs for outreach to MSM and IDUs reflect low current coverage; these were applied throughout 2015–2019 without efficiencies that may be realized through economies of scale [10]. Finally, projections ignored cost variation among regions (e.g. higher logistics and transport cost in the North, where commodities are trucked from Southern harbors instead of directly shipped).

In conclusion, the NSP is an ambitious but sensible strategy for reducing health and economic burdens of HIV in Mozambique. Projections support the feasibility of reducing new infections by 40%, and HIV-related deaths by 30% by 2019 from 2014 numbers, if stated coverage targets for both treatment and behavioural prevention, notably outreach to key groups, can be achieved. The proposed NSP will not turn the epidemic into elimination yet (as aimed for in UNAIDS’ 90-90-90 and Fast Track targets); that will require further scale-up of targeted behavioural prevention and expanded treatment.

## Supporting Information

S1 File *Goals* assumptions on HIV/AIDS natural history. (DOCX)

S2 File *Goals* assumptions on demography, HIV/AIDS risk behaviours and epidemic onset, for three regions of Mozambique. (DOCX)

## References

[1] World Health Organization (2013) Consolidated guidelines on the use of antiretroviral drugs for treating and preventing HIV infection: recommendations for a public health approach. Geneva. 24716260

[2] National Institute of Allergy and Infectious Diseases (NIAID) (2015) Starting Antiretroviral Treatment Early Improves Outcomes for HIV-Infected Individuals (START trial). In: National Institute of Allergy and Infectious Diseases (NIAID), editor. Washington DC.

[3] Danel C, Gabillard D, Le Carrou J, Anglaret X, Moh R, et al. Early ART and IPT in HIV-Infected African Adults with High CD4 Count (Temprano Trial) 2015 23–26 February; Seattle, Washington. pp. Abstract 115LB.

[4] World Health Organization (2011) Joint strategic action framework to accelerate the scale-up of voluntary medical male circumcision for HIV prevention in Eastern and Southern Africa—2012–2016. Geneva.

[5] Republic of Mozambique Conselho de Ministros / Conselho Nacional de Combate ao HIV/SIDA (2010) Plano Estratégico Nacional de Resposta ao HIV e SIDA 2010–2014 (PEN III). Maputo.

[6] Stover J (2004) Projecting the demographic consequences of adult HIV prevalence trends: the Spectrum Projection Package. Sex Transm Infect 80 Suppl 1: i14–18. 15249694 10.1136/sti.2004.010157 PMC1765840

[7] Stover J, Bollinger L, Avila C (2011) Estimating the impact and cost of the WHO 2010 recommendations for antiretroviral therapy. AIDS Research and Treatment Article ID 738271. 10.1155/2011/738271 PMC3066594 21490782

[8] Stover J, Fidzani B, Molomo BC, Moeti T, Musuka G (2008) Estimated HIV trends and program effects in Botswana. PLoS ONE 3: e3729 10.1371/journal.pone.0003729 19008957 PMC2579326

[9] Stover J, Hallett TB, Wu Z, Warren M, Gopalappa C, et al (2014) How Can We Get Close to Zero? The Potential Contribution of Biomedical Prevention and the Investment Framework towards an Effective Response to HIV. PLoS One 9: e111956 10.1371/journal.pone.0111956 25372770 PMC4221192

[10] Stover J, Korenromp EL, Blakley M, Viisainen K, Komatsu R, et al (2011) Long-term costs and health impact of continued Global Fund support for antiretroviral therapy. PLoS ONE 10.1371/journal.pone.0021048 PMC3121720 21731646

[11] Avenir Health (2014) Spectrum Manual, Spectrum System of Policy Models: Resource Needs Model (RNM). In: Avenir Health, editor. Spectrum Manual, Spectrum System of Policy Models. Glastonbury, CT.

[12] Stover J, Bollinger L (2007) Financial resources required to achieve universal access to HIV prevention, treatment, care and support Description of Interventions/Services Included in the Estimates of Resources Needed for HIV and AIDS. Methodological Annex–IV. Glastonbury, CT: Avenir Health.

[13] Bollinger LA (2008) How can we calculate the "E" in "CEA"? AIDS 22 suppl 1: S51–S57. 10.1097/01.aids.0000327623.31856.45 18664954

[14] Avenir Health (2011) Goals manual: a model for estimating the effects of interventions and resource allocation on HIV infections and deaths. Glastonbury, CT.

[15] Bollinger L, Perham KE, DeCormier Plosky W. Grading Goals: a systematic review of studies in the Goals model; 2010 18–23 July; Vienna. pp. Abstract no. WEPE0817.

[16] Cohen MS, Chen YQ, McCauley M, Gamble T, Hosseinipour MC, et al (2011) Prevention of HIV-1 infection with early antiretroviral therapy. N Engl J Med 365: 493–505. 10.1056/NEJMoa1105243 21767103 PMC3200068

[17] Attia S, Egger M, Muller M, Zwahlen M, Low N (2009) Sexual transmission of HIV according to viral load and antiretroviral therapy: systematic review and meta-analysis. AIDS 23: 1397–1404. 10.1097/QAD.0b013e32832b7dca 19381076

[18] Tanser F, Barnighausen T, Grapsa E, Zaidi J, Newell ML (2013) High coverage of ART associated with decline in risk of HIV acquisition in rural KwaZulu-Natal, South Africa. Science 339: 966–971. 10.1126/science.1228160 23430656 PMC4255272

[19] Kingdom of Swaziland: Ministry of Health (2012) Swaziland HIV Incidence Measurement Survey (SHIMS) 2010–2012: First findings report.

[20] Justman J, Ellman T, Donnell D, Duong YT, Reed J, et al. Population HIV viral load estimate in Swaziland: Assessing ART program effectiveness and transmission potential; 2013; 20th Conference on Retroviruses and Opportunistic Infections, Atlanta, GA.

[21] Korenromp EL, Stover J, Gobet B, Fazito E (2015) Impact and Cost of the HIV/AIDS National Strategic Plan in Mozambique, 2015–2019 –projections with the Spectrum/Goals model. Maputo. 10.1371/journal.pone.0142908 PMC4643916 26565696

[22] Grupo tecnico multisectoral de apoio à luta contra o HIV/SIDA em Moçambique (2013) Ronda de Vigilância Epidemiológica do HIV e Sífilis em Moçambique, 2011: Principais Resultados. Maputo.

[23] Instituto Nacional de Estatística MdSM, MEASURE DHS / ICF International (2013) Mozambique Demographic and Health Survey 2011. Calverton, Maryland USA: MEASURE DHS / ICF International.

[24] ICF Macro, Ministry of Health (Mozambique), National Health Institute (Mozambique), National Statistics Institute (Mozambique) (2010) Mozambique AIDS Indicator Survey 2009. Calverton, Maryland, USA: ICF Macro.

[25] Mozambique Instituto Nacional de Saúde (INS), Centros de Controle e Prevenção de Doenças dos EUA (CDC), Universidade de Califórnia SFU, Pathfinder International, Centro Internacional de Formação e Educação para a Saúde (I-TECH) (2013) Inquérito Integrado Biológico e Comportamental entre Mulheres Trabalhadoras de Sexo, Moçambique 2011–2012: Relatório Final. São Francisco: UCSF / Mozambique Ministry of Health.

[26] Mozambique Instituto Nacional de Saúde (INS), Centros de Controle e Prevenção de Doenças dos EUA (CDC), Population Services International (PSI), Universidade de Califórnia SFU, Pathfinder International, et al (2013) Inquérito Integrado, Biológico e Comportamental entre Homens que Fazem Sexo com Homens, Moçambique 2011: Relatório Final. São Francisco: UCSF / Mozambique Ministry of Health.

[27] Mozambique Ministry of health (MISAU), Instituto Nacional de Saúde (INS), Centros de Controle e Prevenção de Doenças dos EUA (CDC), Universidade de Califórnia SFU, Mozambique Ministério do Trabalho (MITRAB), et al (2013) Inquérito Integrado Biológico e Comportamental entre Trabalhadores Moçambicanos nas Minas da República da África do Sul, Moçambique 2012: Relatório Final. Maputo: Mozambique Ministry of Health (MISAU).

[28] Ghani AC, Aral SO (2005) Patterns of sex worker-client contacts and their implications for the persistence of sexually transmitted infections. J Infect Dis 191 Suppl 1: S34–41. 15627229 10.1086/425276

[29] Lafort Y, Geelhoed D, Cumba L, Lazaro C, Delva W, et al (2010) Reproductive health services for populations at high risk of HIV: Performance of a night clinic in Tete province, Mozambique. BMC Health Serv Res 10: 144 10.1186/1472-6963-10-144 20507644 PMC2890643

[30] Amin A, Boothe M, Cassimo MN, Duce P, Fazito E, et al (2013) Distribuição da incidência de infecções por HIV na população de 15 a 49 anos em Moçambique por modo de transmissão, 2013 Maputo: U.S. Agency for International Development (USAID), Center for Diseases Control and Prevention (CDC), Instituto Nacional de Estatística (INE), Programa Conjunto das Nações Unidas para o SIDA (ONUSIDA), University of California San Francisco (UCSF), Conselho Nacional de Combate ao HIV e SIDA (CNCS), Ministério da Saúde (MISAU), Instituto Nacional de Saúde (INS), International Organization for Migration (IOM).

[31] Republic of Mozambique Conselho Nacional de Combate ao HIV e SIDA (CNCS) / National AIDS Council (2012) Global AIDS Response Progress Report for the period 2010–2011. Maputo.

[32] Republic of Mozambique Conselho Nacional de Combate ao HIV e SIDA (CNCS) / National AIDS Council (2014) Global AIDS Response Progress Report. Maputo.

[33] Figueroa ME, Kincaid DL, Hurley EA (2014) The effect of a joint communication campaign on multiple sex partners in Mozambique: the role of psychosocial/ideational factors. AIDS Care 26 Suppl 1: S50–55. 10.1080/09540121.2014.907386 24749940

[34] Republic of Mozambique Ministry of Health (2013) Plano de Aceleraçao da resposta ao HIV é SIDA em Moçambique 2013–2015 / HIV/AIDS Acceleration Plan. Maputo.

[35] Republica de Moçambique Ministério dau Saude Direcçao Nacional de Saude Publica Departamento de Saude da mulher e da criança (2012) National plan to eliminate Mother-to-Child Transmission of HIV, 2012–2015.

[36] Country Coordinating Mechanism of Mozambique (2014) Global Fund TB and HIV concept note for 2015–2017. Maputo.

[37] Dutta A, Perales N, Silva R, Cirera i Crivillé L (2014) Estimated Resource Needs and Impact of Mozambique’s Plano Estratégico do Sector Saúde, 2014–2019—applying the One Health Tool. Futures Group, Health Policy Project.

[38] Mozambique Ministério da Saúde (2014) Relatório Semestral 2014 das Actividades relacionadas ao HIV/SIDA. Maputo.

[39] Mahy M, Lewden C, Brinkhof MW, Dabis F, Tassie JM, et al (2010) Derivation of parameters used in Spectrum for eligibility for antiretroviral therapy and survival on antiretroviral therapy. Sex Transm Infect 86 Suppl 2: ii28–34. 10.1136/sti.2010.044255 21106512 PMC3173808

[40] Stover J, Brown T, Marston M (2012) Updates to the Spectrum/Estimation and Projection Package (EPP) model to estimate HIV trends for adults and children. Sex Transm Infect 88 Suppl 2: i11–16. 10.1136/sextrans-2012-050640 23172341 PMC3512426

[41] Mozambique Ministério da Saúde (2013) Plano Estratégico do Sector da Saúde (PESS) 2013–2017. Maputo.

[42] UNAIDS Mozambique (2011) Mediçao de gastos em SIDA (MEGAS) para o periodo: 2010–2011. Nivel e fluxo de recursos e despesas para a resposta nacional ao HIV e SIDA. Maputo.

[43] United States President’s Emergency Plan for Aids Relief (2015) Supply Chain Management System. Washington DC.

[44] Avenir Health (2014) Avenir Health HIV Unit Cost Repository. December ed. Glastonbury (CT).

[45] Conselho Nacional de Combate ao SIDA (CNCS), UNAIDS (2014) Medição de Gastos em SIDA, 2010 & 2011 [National AIDS Spending Assessment]. Maputo.

[46] UNAIDS (2012) Key Programmes to Reduce Stigma and Discrimination and Increase Access to Justice In National HIV Responses. Geneva.

[47] World Bank World Development Indicators database.

[48] Granich R, Kahn JG, Bennett R, Holmes CB, Garg N, et al (2012) Expanding ART for Treatment and Prevention of HIV in South Africa: Estimated Cost and Cost-Effectiveness 2011–2050. PLoS ONE 7: e30216 10.1371/journal.pone.0030216 22348000 PMC3278413

[49] Resch S, Korenromp EL, Stover J, Blakley M, Krubiner C, et al (2011) Economic returns to Investment in AIDS treatment in low- and middle-income countries. PLoS ONE 6: e25310 10.1371/journal.pone.0025310 21998648 PMC3187775

[50] UNAIDS (2014) Fast-Track: ending the AIDS epidemic by 2030 Geneva.

[51] World Health Organization (2014) Global Health Expenditure Database. 2014.

[52] Ruperez M, Pou C, Maculuve S, Cedeno S, Luis L, et al (2015) Determinants of virological failure and antiretroviral drug resistance in Mozambique. J Antimicrob Chemother. 10.1093/jac/dkv143 26084302

[53] Stoneburner R, Korenromp EL, Lazenby M, Tassie JM, Letebele J, et al (2014) Using health surveillance data to assess the impact of AIDS and antiretroviral treatment on adult mortality and morbidity in Botswana. PLoS One 9: e100431 10.1371/journal.pone.0100431 25003870 PMC4086724

[54] van Schalkwyk C, Mndzebele S, Hlophe T, Garcia Calleja JM, Korenromp EL, et al (2013) Outcomes and Impact of HIV Prevention, ART and TB Programs in Swaziland—Early Evidence from Public Health Triangulation. PLoS One 8: e69437 10.1371/journal.pone.0069437 23922711 PMC3724860

[55] Korenromp EL, Bakker R, Gray R, Wawer MJ, Serwadda D, et al (2002) The effect of HIV, behavioural change, and STD syndromic management on STD epidemiology in sub-Saharan Africa: simulations of Uganda. Sex Transm Infect 78 Suppl 1: i55–63. 12083448 10.1136/sti.78.suppl_1.i55 PMC1765831

