# Supplementary material for: Impact and Cost of the HIV/AIDS National Strategic Plan for Mozambique, 2015-2019—Projections with the Spectrum/Goals Model
Source: PLoS One. 2015 Nov 13;10(11):e0142908. doi: 10.1371/journal.pone.0142908 (PMC4643916; doi:10.1371/journal.pone.0142908)
Supplement: S1 File — (DOCX) [file pone.0142908.s001.docx]

**S1 file. *Goals* assumptions on HIV/AIDS natural history.**

| **Parameter** | **Value** |
| --- | --- |
| HIV transmission probability, per act (female to male) | 0.0010 in North, 0.0014 in Center & South [[1](#_ENREF_1),[2](#_ENREF_2)] |
| Multiplier on transmission per act for:   - Male to female - Presence of STI - MSM contact | 1.0 [[3](#_ENREF_3)]  6.2 for North, 7.0 for Center, 6.6 for South [[4](#_ENREF_4),[5](#_ENREF_5)]  2.6 [[6](#_ENREF_6)] |
| Relative infectiousness, by stage of HIV infection   - Primary infection (3 months) - Asymptomatic - Symptomatic - On ART | 15 in North, 20 in Center, 16 in South [[7](#_ENREF_7),[8](#_ENREF_8)].  1.0, as reference stage.  3.75 [[9-13](#_ENREF_9)].  0.75, i.e. an 80% reduced infectivity compared to the symptomatic stage if untreated [[9-13](#_ENREF_9)]. |
| ART survival effect | *Goals* default Southern Africa regional pattern [[14](#_ENREF_14)], except for patients on ART for >12 months, where we assumed 20% additional mortality to account for relatively high drop-out from ART in Mozambique [[15](#_ENREF_15)] |
| Efficacy in reducing HIV transmission   - Condom use - Male circumcision | 0.8 [[16](#_ENREF_16)]  0.6 [[17-19](#_ENREF_17)] |

**References for S1 File:**

1. Baggaley RF, Fraser C (2010) Modelling sexual transmission of HIV: testing the assumptions, validating the predictions. *Curr Opin HIV AIDS* 5: 4, 269-276.

2. Gray RH, Wawer MJ (2012) Probability of heterosexual HIV-1 transmission per coital act in sub-Saharan Africa. *J Infect Dis* 205: 3, 351-352.

3. Galvin SR, Cohen MS (2004) The role of sexually transmitted diseases in HIV transmission. *Nat Rev Microbiol* 2: 1, 33-42.

4. Powers KA, Poole C, Pettifor AE, Cohen MS (2008) Rethinking the heterosexual infectivity of HIV-1: a systematic review and meta-analysis. *Lancet Infect Dis* 8: 9, 553-563.

5. Korenromp EL, de Vlas SJ, Nagelkerke NJ, Habbema JD (2001) Estimating the magnitude of STD cofactor effects on HIV transmission: how well can it be done? *Sex Transm Dis* 28: 11, 613-621.

6. Vittinghoff E, Douglas J, Judson F, McKirnan D, MacQueen K, et al. (1999) Per-contact risk of human immunodeficiency virus transmission between male sexual partners. *Am J Epidemiol* 150: 3, 306-311.

7. Boily MC, Baggaley RF, Wang L, Masse B, White RG, et al. (2009) Heterosexual risk of HIV-1 infection per sexual act: systematic review and meta-analysis of observational studies. *Lancet Infect Dis* 9: 2, 118-129.

8. Pinkerton SD (2008) Probability of HIV transmission during acute infection in Rakai, Uganda. *AIDS Behav* 12: 5, 677-684.

9. Cohen MS, Chen YQ, McCauley M, Gamble T, Hosseinipour MC, et al. (2011) Prevention of HIV-1 infection with early antiretroviral therapy. *N Engl J Med* 365: 6, 493-505.

10. Attia S, Egger M, Muller M, Zwahlen M, Low N (2009) Sexual transmission of HIV according to viral load and antiretroviral therapy: systematic review and meta-analysis. *AIDS* 23: 11, 1397-1404.

11. Tanser F, Barnighausen T, Grapsa E, Zaidi J, Newell ML (2013) High coverage of ART associated with decline in risk of HIV acquisition in rural KwaZulu-Natal, South Africa. *Science* 339: 6122, 966-971.

12. Kingdom of Swaziland: Ministry of Health 2012. Swaziland HIV Incidence Measurement Survey (SHIMS) 2010-2012: First findings report.

13. Justman J, Ellman T, D. D, al. e. Population HIV viral load estimate in Swaziland: Assessing ART program effectiveness and transmission potential; 2013; Atlanta, GA.

14. Stover J, Hallett TB, Wu Z, Warren M, Gopalappa C, et al. (2014) How Can We Get Close to Zero? The Potential Contribution of Biomedical Prevention and the Investment Framework towards an Effective Response to HIV. *PLoS One* 9: 11, e111956.

15. Republic of Mozambique Conselho Nacional de Combate ao HIV e SIDA (CNCS) / National AIDS Council 31 March 2014. Global AIDS Response Progress Report. Maputo. <http://www.unaids.org/sites/default/files/country/documents/MOZ_narrative_report_2014.pdf>.

16. Weller S, Davis K (2002) Condom effectiveness in reducing heterosexual HIV transmission. *Cochrane Database Syst Rev*: 1, CD003255.

17. Auvert B, Puren A, Taljaard D, Lagarde E, Tambekou-Sobngwi J, et al. The impact of male circumcision on the female-to-male transmission of HIV: results of the intervention trial ANRS 1265; 2005; Paris, France. INSERM.

18. Gray RH, Li X, Kigozi G, Serwadda D, Nalugoda F, et al. (2007) The impact of male circumcision on HIV incidence and cost per infection prevented: a stochastic simulation model from Rakai, Uganda. *Aids* 21: 7, 845-850.

19. Bailey RC, Moses S, Parker CB, Agot K, Maclean I, et al. (2007) Male circumcision for HIV prevention in young men in Kisumu, Kenya: a randomised controlled trial. *Lancet* 369: 9562, 643-656.
