# Supplementary material for: Impact and Cost of the HIV/AIDS National Strategic Plan for Mozambique, 2015-2019—Projections with the Spectrum/Goals Model
Source: PLoS One. 2015 Nov 13;10(11):e0142908. doi: 10.1371/journal.pone.0142908 (PMC4643916; doi:10.1371/journal.pone.0142908)
Supplement: S2 File — (DOCX) [file pone.0142908.s002.docx]

**S2 File. *Goals* assumptions on demography, HIV/AIDS risk behaviours and epidemic onset, for three regions of Mozambique**

| **Parameter** | **North** |  | **Central** |  | **South** |  | **Sources / explanation** |
| --- | --- | --- | --- | --- | --- | --- | --- |
|  | **Men** | **Women** | **Men** | **Women** | **Men** | **Women** |  |
| Population: |  |  |  |  |  |  |  |
| Not sexually active / never had sex | 3.7% | 16% | 13% | 21% | 11% | 18% | DHS. AIS, IBBS [[1-5](#_ENREF_1)] |
| Low-risk heterosexual / one partner in last year | 46% | 57% | 57% | 51% | 30% | 50% |  |
| Medium-risk heterosexual (>1 partner in last year) | 26% | 24% | 20% | 24% | 47% | 30% |  |
| High-risk heterosexual: female sex worker or client | 22% | 3.6% | 8.3% | 3.8% | 10% | 2.4% |  |
| IDU | 0.10% | 0.01% | 0.09% | 0.01% | 0.17% | 0.01% |  |
| MSM | 1.6% |  | 1.5% |  | 1.3% |  |  |
| Proportion married or in stable union: |  |  |  |  |  |  |  |
| Low-risk heterosexual / one partner in last year | 100% | 100% | 100% | 100% | 100% | 100% | DHS. AIS, IBBS [[1-5](#_ENREF_1)] |
| Medium-risk heterosexual (>1 partner in last year) | 51% | 49% | 52% | 56% | 40% | 36% |  |
| High-risk heterosexual: female sex worker or client | 35% | 11% | 37% | 4.4% | 30% | 4.6% |  |
| IDU | 0% | 0% | 0% | 0% | 0% | 0% |  |
| MSM | 7.4% |  | 7.6% |  | 6.8% |  |  |
| Age at first sex (years) | 16.4 | 15.6 | 17.2 | 16.2 | 18.3 | 16.5 | DHS, AIS [[1](#_ENREF_1),[2](#_ENREF_2)] |
| Start of the HIV epidemic | 1989 |  | 1985 |  | 1988 |  | ANC surveillance [[6](#_ENREF_6)] |

**References for S2 File:**

1. Instituto Nacional de Estatística MdSM, MEASURE DHS / ICF International. March 2013. Mozambique Demographic and Health Survey 2011. Calverton, Maryland USA: MEASURE DHS / ICF International. <http://dhsprogram.com/pubs/pdf/FR266/FR266.pdf>.

2. ICF Macro, Ministry of Health (Mozambique), National Health Institute (Mozambique), National Statistics Institute (Mozambique). 2010. Mozambique AIDS Indicator Survey 2009. Calverton, Maryland, USA: ICF Macro. <http://dhsprogram.com/publications/publication-AIS8-AIS-Final-Reports.cfm#sthash.SpRHafiZ.dpuf>.

3. Mozambique Instituto Nacional de Saúde (INS), Centros de Controle e Prevenção de Doenças dos EUA (CDC), Universidade de Califórnia SFU, Pathfinder International, Centro Internacional de Formação e Educação para a Saúde (I-TECH). October 2013. Inquérito Integrado Biológico e Comportamental entre Mulheres Trabalhadoras de Sexo, Moçambique 2011–2012: Relatório Final. São Francisco: UCSF / Mozambique Ministry of Health.

4. Mozambique Instituto Nacional de Saúde (INS), Centros de Controle e Prevenção de Doenças dos EUA (CDC), Population Services International (PSI), Universidade de Califórnia SFU, Pathfinder International, et al. October 2013. Inquérito Integrado, Biológico e Comportamental entre Homens que Fazem Sexo com Homens, Moçambique 2011: Relatório Final. São Francisco: UCSF / Mozambique Ministry of Health.

5. Mozambique Ministry of health (MISAU), Instituto Nacional de Saúde (INS), Centros de Controle e Prevenção de Doenças dos EUA (CDC), Universidade de Califórnia SFU, Mozambique Ministério do Trabalho (MITRAB), et al. October 2013. Inquérito Integrado Biológico e Comportamental entre Trabalhadores Moçambicanos nas Minas da República da África do Sul, Moçambique 2012: Relatório Final. Maputo: Mozambique Ministry of Health (MISAU).

6. Grupo tecnico multisectoral de apoio à luta contra o HIV/SIDA em Moçambique October 2013. Ronda de Vigilância Epidemiológica do HIV e Sífilis em Moçambique, 2011: Principais Resultados. Maputo.
